# Lung Fibroblasts Share Mesenchymal Stem Cell Features Which Are Altered in Chronic Obstructive Pulmonary Disease via the Overactivation of the Hedgehog Signaling Pathway

**DOI:** 10.1371/journal.pone.0121579

**Published:** 2015-03-27

**Authors:** Florence Figeac, Maylis Dagouassat, Meriem Mahrouf-Yorgov, Sabine Le Gouvello, Céline Trébeau, Angeliqua Sayed, Jean-Baptiste Stern, Pierre Validire, Jean-Luc Dubois-Randé, Jorge Boczkowski, Isabelle Mus-Veteau, Anne-Marie Rodriguez

**Affiliations:** 1 Inserm, U955, Equipe 12, Créteil, 94000, France; 2 Inserm, U955, Equipe 4, Créteil, 94000, France; 3 Université Paris Est, Faculté de Médecine, Créteil, 94000, France; 4 AP-HP, Hôpital Henri Mondor- A. Chenevier, Département d’Hématologie et Immunologie Biologiques, Créteil, 94000, France; 5 Institut Mutualiste Montsouris, Département thoracique, Paris, France; 6 Institut Mutualiste Montsouris, Département anatomopathologie, Paris, France; 7 AP-HP, Hôpital Henri Mondor-A. Chenevier, Service de Physiologie Explorations Fonctionnelles, Créteil, 94000, France; 8 Inserm, U955, Equipe 3, Créteil, 94000, France; 9 Hôpital Intercommunal de Créteil, Service de pneumologie et pathologie professionnelle, Créteil, 94000, France; 10 Institut de Pharmacologie Moléculaire et Cellulaire CNRS, Valbonne 06560, France; 11 Université de Nice-Sophia-Antipolis, Nice 06108, France; Institut Curie, FRANCE

## Abstract

**Background:**

Alteration of functional regenerative properties of parenchymal lung fibroblasts is widely proposed as a pathogenic mechanism for chronic obstructive pulmonary disease (COPD). However, what these functions are and how they are impaired in COPD remain poorly understood. Apart from the role of fibroblasts in producing extracellular matrix, recent studies in organs different from the lung suggest that such cells might contribute to repair processes by acting like mesenchymal stem cells. In addition, several reports sustain that the Hedgehog pathway is altered in COPD patients thus aggravating the disease. Nevertheless, whether this pathway is dysregulated in COPD fibroblasts remains unknown.

**Objectives and Methods:**

We investigated the stem cell features and the expression of Hedgehog components in human lung fibroblasts isolated from histologically-normal parenchymal tissue from 25 patients—8 non-smokers/non-COPD, 8 smokers-non COPD and 9 smokers with COPD—who were undergoing surgery for lung tumor resection.

**Results:**

We found that lung fibroblasts resemble mesenchymal stem cells in terms of cell surface marker expression, differentiation ability and immunosuppressive potential and that these properties were altered in lung fibroblasts from smokers and even more in COPD patients. Furthermore, we showed that some of these phenotypic changes can be explained by an over activation of the Hedgehog signaling in smoker and COPD fibroblasts.

**Conclusions:**

Our study reveals that lung fibroblasts possess mesenchymal stem cell-features which are impaired in COPD via the contribution of an abnormal Hedgehog signaling. These processes should constitute a novel pathomechanism accounting for disease occurrence and progression.

## Introduction

Chronic obstructive pulmonary disease (COPD) is an incurable disease representing the fourth leading cause of death worldwide. It is characterized by a heterogeneous collection of conditions associated with chronic expiratory airflow reduction and high risk of lung cancer. Exposure to tobacco smoke is the main cause of COPD and accounts for up to 80% of cases. However, only around 20% of smokers are at risk for the disease [1]. This observation reflects differences in effectiveness of lung repair mechanisms between the smokers who develop COPD in response to smoke-induced injuries and those who do not.

Impairment of functional properties of lung fibroblasts, which are key players in maintaining tissue homeostasis, is believed to be an important mechanism underlying COPD. Several processes accounting for fibroblast dysfunction have been described such as activation of both apoptosis and nucleic acid oxidation, inhibition of fibroblast proliferation and fibronectin synthesis, loss of contractile properties, deregulation of soluble factor secretion and senescence [1–7].

Interestingly, several recent studies conducted in various organs have shown that fibroblasts share some features with mesenchymal stem cells (MSC). In particular, fibroblasts exhibit cell-surface expression pattern similar to MSC [8,9], are capable of multilineage differentiation into adipocytes, osteoblasts and chondrocytes [8,10] and display immunosuppressive properties [11]. Whether these cells could repair tissue damage through modalities classically attributed to MSC [10] remains an open question and, although the physiological significance of these MSC-like features in fibroblasts is still unknown, it is tempting to suggest that alteration of these properties is involved in the development and/or progression of several degenerative diseases. This hypothesis may also apply to COPD but specific studies comparing lung parenchyma fibroblasts to MSC have never been carried out. An important signaling pathway that could influence COPD progression or exacerbation is Hedgehog (Hh). In fact, evidences from microarray profiling and genome-wide association studies in human subjects and animal models suggest that this pathway is disturbed in COPD [12]. Hh signaling pathway has been shown to orchestrate lung organogenesis and plays during the adult life a central role in tissue repair [13], cancer development [14] and stem cell fate [15–19]. Hh signaling is initiated by the binding of the Hh ligand to its transmembrane receptor Patched1 (Ptc), relieving suppression of the transmembrane protein Smoothened (Smo). Smo activates an intracellular cascade that results in activation of Gli transcription factors which mediate Hh specific responses in the cell by modulating gene expression [20]. Intriguingly, two independent studies in COPD patients have reported a mutation near the locus of Hh interacting protein (Hhip) [21,22], which is a critical regulator of the Hh pathway [23]. However, whether Hh signaling is dysregulated in fibroblasts from COPD patients remains unknown.

Here, we investigated stemness properties and Hh signaling in human adult lung parenchymal fibroblasts from non smokers, smokers and COPD patients.

## Material and Methods

### Clinical features of lung fibroblast donors

Lung fibroblasts were obtained from 25 patients: 8 non-smokers (C-NS), 8 smokers (C-S) without clinical or functional signs of COPD and 9 smokers with mild and moderate COPD (COPD) according to the Global Initiative for Chronic Obstructive Lung Disease classification (http://www.goldcopd.org/). The three groups were closely matched in age and sex and the two categories of smokers, with or without COPD, had similar smoking history (see clinical features in Table 1). As expected, patients with COPD showed lung functional alterations, including significantly lower forced expiratory volume in one sec (FEV_1_) compared to controls. All COPD patients had pulmonary emphysema as assessed by CT scan. Histology of lung tumors was similar among the three groups (data not shown). None of the patients had chronic cardiovascular, hepatic or renal disease or had undergone chemotherapy for cancer.

**Table 1:** Subject characteristics.

	**Non-smokers controls**	**Smokers controls**	**COPD patients**	**p**
**Patient (no.)**	8	8	9	
**Age (years)**	66 (42–80)	59 (51–68)	58 (51–62)	NS
**Sex (M/F)**	3/5	3/5	4/5	
**FEV**_**1**_%	99 (87–110)	86 (69–102)	$ 70 (57–77	p<0.05
**FEV**_**1**_ **/VC %**	83 (80–100)	78 (71–85)	*/** 61 (42–69)	p<0.01
**GOLD classification 0/I/II/III/IV**	8/0/0/0/0	8/0/0/0/0	0/3/6/0/0	
**Smoking history**	0	56 (23–100)	48 (30–60)	NS

Data are expressed as median and minimum and maximum quartiles (in parentheses) $ p<0.05 COPD patients vs. non smokers and smokers

*p<0.05 COPD patients vs. smokers.

** p< 0.01 COPD patients vs. non-smokers.

VC, vital capacity; FEV_1_, forced expiratory volume in 1 sec; COPD, chronic obstructive pulmonary disease; GOLD, Global Initiative for Chronic Obstructive Lung Disease classification

### Cell isolation and cell culture conditions

Lung fibroblasts were isolated from lung tissue of patients undergoing resective surgery for pulmonary carcinoma. After careful macroscopic evaluation, pleura-free parenchymal specimens were excised from peripheral areas of the lobe as far away from the tumor site as possible. Dermal fibroblasts were isolated from healthy donor skin biopsies. Lung and dermal fibroblasts were obtained by the explant method as previously described [24,25]. Human Multipotent Adipose Derived stem cells (hMADS cells) were used as MSC model, regarding their high capacity to proliferate and differentiate into several specialized cells. These cells were isolated and expanded as previously reported [26]. Written informed consent was obtained from patients and the study was approved by the “Comité de Protection des Personnes Ile de France IX”. All participants provided their written informed consent to participate in this study.

All cell types were cultured in Dulbecco’s modified Eagle’s medium (DMEM), 1 g/L glucose containing 10% heat-inactivated fetal bovine serum (FBS) (Dominique Dutscher, Brumath, France), 100 U/ml penicillin, 100 μg/ml streptomycin, and 10 mM HEPES (Invitrogen, Cergy-Pontoise, France), at 37°C in a 5% CO_2_ atmosphere.

All experiments were performed using lung fibroblasts at passage 3.

### RT-PCR

RNA was extracted with the Quiagen Rneasy Mini Kit (Qiagen, Courtaboeuf, France) and then reverse-transcribed using the Superscript First-Strand Synthesis System (Invitrogen, Villebon sur Yvette, France) and Oligo(dT)20. Quantitative RT-PCR reactions were performed in triplicate on a 7900 real-time PCR detection system (Applied Biosystems) using Platinium SYBR Green qPCR SuperMix (Invitrogen). PCR conditions were 50°C for 2 min, 95°C for 2 min, 45 cycles at 95°C for 15s, and 60°C for 45s, using GAPDH or SF3A1 as the reference gene. Primers sequences are described in Table 2.

**Table 2:** Primer sequences used for qPCR assays.

**Human genes**	**Forward**	**Reverse**
**p16**	GGGTCGGGTAGAGGAGGTG	CATCATGACCTGGATCGGC
**p53**	CCTGAGGTTGGCTCTGACTGTA	TGTTCCGTCCCAGTAGATTACCA
**SF3A1**	TGCAGGATAAGACGGAATGGAAACTGA	GTAGTAAGCCAGTGAGTTGGAATCTTTG
**ALP**	CTGTGGGCATTGTGACC	TGTATTTCCGGCCACC
**Osteocalcin**	CCTTTGTGTCCAAGCAGG	CACAGTCCGGATTGAGC
**SOX9**	GCGGCGGAGGAAGTC	TGGGTGGGGTCGGTG
**Aggrecan**	CATTTGCCAGGGGGGGT	GGGGTATCTGACGGTC
**PPAR γ**	GCTGAATCCAGAGTCCG	GGGGGTGATGTGTTTGAAC
**LPL**	CAGCCTCCGGCTCAG	GGAGGCGGTCAGACT
**MMP1**	CTTGCACTGAGAAAGAAGACAAAGG	ACACCCCAGAACAGCAGCA
**IGF2**	GACACCCTCCAGTTCGTCTG	CGGAAACAGCACTCCTCAAC
**GLI1**	TCCTTACCTCCCAACCTCTGTCTAC	GTCCATATAGGGGTTCAGACCACTG
**GLI2**	GCCTCCGAGAAGCAAGAAGCCAAAA	CCTGGTGTCGCATGTCAATCGGTAG
**GLI3**	CGGGACGGTGTTTGCCATGGAC	GGAGGATGGAAGGCAGGGAAAAGAT
**PATCHED**	AAGTATATGCACTGGCAGGAGGAG	GTGTGAGACATACTCGTACCCCTTG
**SMOTHENED**	AAGACCTCCTACTTCCACCTGCTC	CACGGTATCGGTAGTTCTTGTAGCC
**α-SMA**	GAAGAGCATCCCACCCTGC	ATTTTCTCCCGGTTGGCCT
**GAPDH**	GCTCTCTGCTCCTCCTGTTC	ACTCCGACCTTCACCTTCC

### **Colony forming unit (CFU) assay, cell proliferation and staining for senescence-associated** β**-galactosidase (SA-**β**-Gal)**

For assessment of CFU, cells were plated at a density of 1000 cells in a 60-mm culture dish. After 14 days, colonies displaying more than 50 cells were counted using Crystal Violet staining (Sigma-Aldrich). Cumulative population doubling levels (PDLs) and staining for SA-β-Gal activity were performed as previously described [24,27].

### Flow cytometry antibodies

Phycoerythrin (PE)-conjugated mouse monoclonal antibodies against human CD117 (clone YB5.B8), CD34 (clone581/CD34), CD45 (clone TU116), CD90 (clone 5E10), CD133 (clone 170411), CD166 (clone 3A6), VEGF-R (clone 89106), CD13 (clone WM15), HLA I (clone G46-2.6), CD15(clone H198), HLA-DR (clone G46-6) and CD44 (clone G44-26) and isotype controls were from R&D Systems (Lille, France) and PE-conjugated CD105 (clone 166707) was from BD Biosciences (Erembodegem, Belgium). Primary unconjugated STRO-1 (clone STRO-1) and secondary fluorescein isothiocyanate (FITC)-goat anti-mouse IgG/IgM antibodies were from R&D Systems and BD Biosciences, respectively. Antibodies were used at the concentration recommended by the manufacturers.

### Cell plasticity

For myofibroblast differentiation, lung fibroblasts were treated with 10 ng/ml TGFβ1 for 3, 4 and 5 days in presence of culture medium containing 1% FBS, prior to staining with a mouse monoclonal antibody against α-smooth muscle actin (α-SMA) (clone 1A4, 1:200, Sigma-Aldrich, Saint-Quentin Fallavier, France) followed by a rhodamine-conjugated secondary antibody (Invitrogen, Saint Aubin, France). Differentiation into adipocytes and osteoblasts was performed as previously described [26]. Chondrogenic differentiation was carried out using Mesenchymal Stem Cell Chondrogenic Differentiation Medium (PromoCell, Heidelberg, Germany).

### Assessment of lung fibroblast paracrine action on peripheral blood monocyte cells (PBMC) proliferation

10^5^ peripheral blood monocyte cells (PBMC) from healthy consenting donors were stimulated with phytohemagglutinin (PHA, 1μg/ml, Invitrogen) and exposed to supernatant of 24-hours cultured cells initially seeded at 4.10^4^cells/ml. After 4 days-exposure, PBMC were overnight exposed to 1μCi ^3^H-thymidine (Perkin-Elmer, Groningen, The Netherlands) prior to measure incorporated radioactivity (TopCount, Perkin-Elmer, Meriden, CT, USA).

In other experiments, 10^5^ PBMC were stimulated with anti-CD3 (clone UCHT1, 2μg/ml, Beckman Coulter, Villepinte, France) and anti-CD28 (clone CD28.2, 2μg/ml, Beckman Coulter) antibodies and labeled with CFSE (1μM, Invitrogen). Stimulated PBMC were then exposed to supernatants collected from fibroblasts. After 4 days, the decrease in CFSE fluorescence assessed by flow cytometry was used to determine PBMC proliferation.

### Treatment with Hedgehog pathway modulators

Prior to differentiation, flow cytometry and immunosuppressive assays, fibroblasts were treated for 48 hours with 10μM Cyclopamine (LC Laboratories, MA, France), 100nM SAG (Merck Millipore), 5 μM Gant 61 (Merck Millipore) or 5μM Purmorphamine (Enzo Life Sciences, Villeurbanne, France). The concentrations of Hh signaling modulators and the treatment duration have been optimized on NIH3T3 fibroblasts in previous studies [28,29]. At these concentrations, drugs were found not cytotoxic as assessed by MTT assay (data not shown). As drugs were solubilized in DMSO, control cells were incubated with equivalent DMSO volumes.

### Immunoblot analysis

Western blotting was performed using standard techniques. Polyclonal anti-human Patched, anti-human Smo and anti-Shh were generously provided by Dr. Martial Ruat (CNRS-UPR 9040, Gif sur Yvette, France) and used at 1/1000 [28,29]. Rabbit polyclonal anti-human Gli1 (C68H3, 1/1000) was purchased from Cell Signaling Technology [30] and monoclonal anti-β tubulin clone TUB 2.1 (1/1000) from SIGMA. Anti-mouse (1/5000) and anti-rabbit (1/2000) horseradish peroxidase conjugated secondary-antibodies came from Dako. Detection was carried out with an ECL kit (Millipore) and a Las3000 imager (Fugi). Patched, Gli1, ShhN, Smo and β-tubulin signals were quantified using Image J software and the intensities of Patched, Gli1, ShhN and Smo signals were corrected for the loading variations using the β-tubulin signals for each patient. Histograms represent the mean Patched, Gli1, ShhN or Smo signal intensity for each patient group from at least 3 independent experiments.

### Primary cilia immunodetection

Primary cilia detection in cultured lung fibroblast was performed as previously described [31–33]. Briefly, fibroblasts were grown to confluence and then switched to medium containing 1% FBS for 48 h. Then, cells were fixed with 4% of paraformaldehyde and permeabilized with 0.05% Triton x-100 (Sigma, Saint Quentin Fallavier, France) prior to be with a mouse anti-acetylated tubulin (1:1000, Sigma Aldrich) primary antibodies, overnight at 4°C. Fluorescence signal was detected by using a goat anti- mouse secondary antibody (1:500, conjugated with Alexa 594, Invitrogen, Cergy-Pontoise, France). The slides were mounted by using prolong DAPI (Invitrogen, Cergy-Pontoise, France) and examined with a fluorescence microscope coupled to a digital camera utilizing axiovision software for image acquisition (Zeiss, Jena, Germany).

### Statistical analysis

Data were analyzed with GraphPad Prism 4.0 (La Jolla, CA, USA) and expressed as mean ± SEM. Comparisons between groups were performed with Kruskall–Wallis’ non-parametric analysis of variance test followed by two-by-two comparisons with Mann–Whitney’s U test when a difference was detected. Comparisons between cells with drug treatment or not in each group were performed with paired t test. p < 0.05 was considered statistically significant.

## Results

### Basic characteristics of lung primary cultured fibroblasts

**Fig 1:**
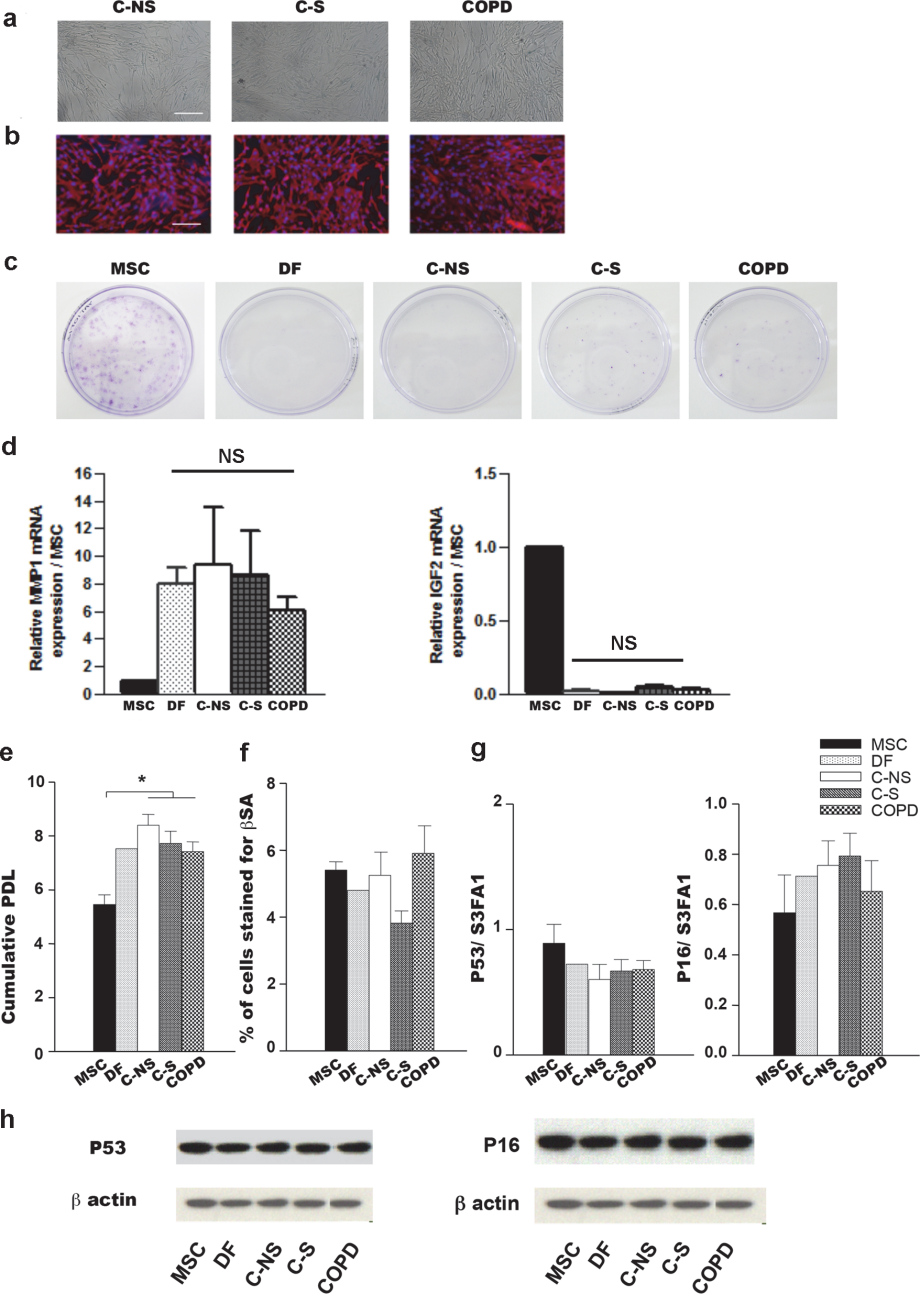
Characterization of lung fibroblast primary cultures. (a) Morphology and (b) vimentin staining (red signal) of lung fibroblasts from C-NS, CS and COPD subjects. Nuclei were counterstained with DAPI (blue signal) Scale bar, 20 μm. (c) CFU assays revealed by crystal violet staining. (d)Relative quantitative transcriptional expression of MMP1 and IGF2 in lung fibroblasts and DF by reference to MSC. (e) Cumulative population doubling level (PDL) and (f) senescence-associated β-galactosidase activity (β-SA) of MSC, DF and lung fibroblasts. (g)Quantitative real-time PCR analysis of mRNA expression of p53 and p16. (h) Protein expression of p53 and p16 by Western blot analysis. (a-h)Results represent means ± SEM of at least 3 independent experiments made with the fibroblast primary cultures at passage 3. NS, non significant; MSC, mesenchymal stem cells; DF, dermal fibroblast; C-NS, non-smoker controls; C-S, smoker controls; COPD, smokers with COPD.

Although lung primary cultures from C-NS, C-S and COPD patients exhibited typical fibroblast morphology and expressed the fibroblastic marker vimentin (Fig 1A and 1B)), we ensured that they were not contaminated by other cell types which could interfere with the interpretation of data. We first examined their colony forming (CFU) ability and their transcriptional expression of MMP1 and IGF2 to distinguish fibroblasts from lung parenchymal MSC. Indeed, MSC have been reported to have greater CFU potential [9] and to express lesser amount of MMP1 but higher level of IGF2 than fibroblasts [34]. Like dermal fibroblasts, lung primary cultures had no or extremely poor ability to form colonies (Fig 1C) and expressed 7 to 9 fold higher levels of MMP1 transcripts and 135 fold lower levels of IGF2 than control MSC (Fig 1D). Lung primary cultures were also found to be devoid of epithelial and endothelial cells as revealed by negative immunostaining for pancytokeratin and CD31, respectively and did not express cancer associated fibroblasts (CAF) markers such as desmin, fibroblast activated protein α and PDGF receptor β (data not shown) confirming the lack of cancer impact. In addition, because replicative senescence has been reported to affect stemness features [35], we analyzed proliferative potential, β-galactosidase activity and expression of senescence markers such as p16 and p53 in the 3 groups of lung fibroblasts (Fig 1E and 1H). We found that whatever the group, these cells had similar proliferative potential (Fig 1E) and were non senescent (Fig 1F and 1H). Interestingly and consistent with previous observations [36], we showed that lung fibroblasts similarly to that of dermis divided faster than did control MSC (Fig 1E). Altogether, these results indicate that the lung fibroblast primary cultures used in this study were equivalent in terms of purity, proliferation and viability.

### Multilineage differentiation potential of lung fibroblasts and their alteration in smoker controls and COPD patients

**Fig 2:**
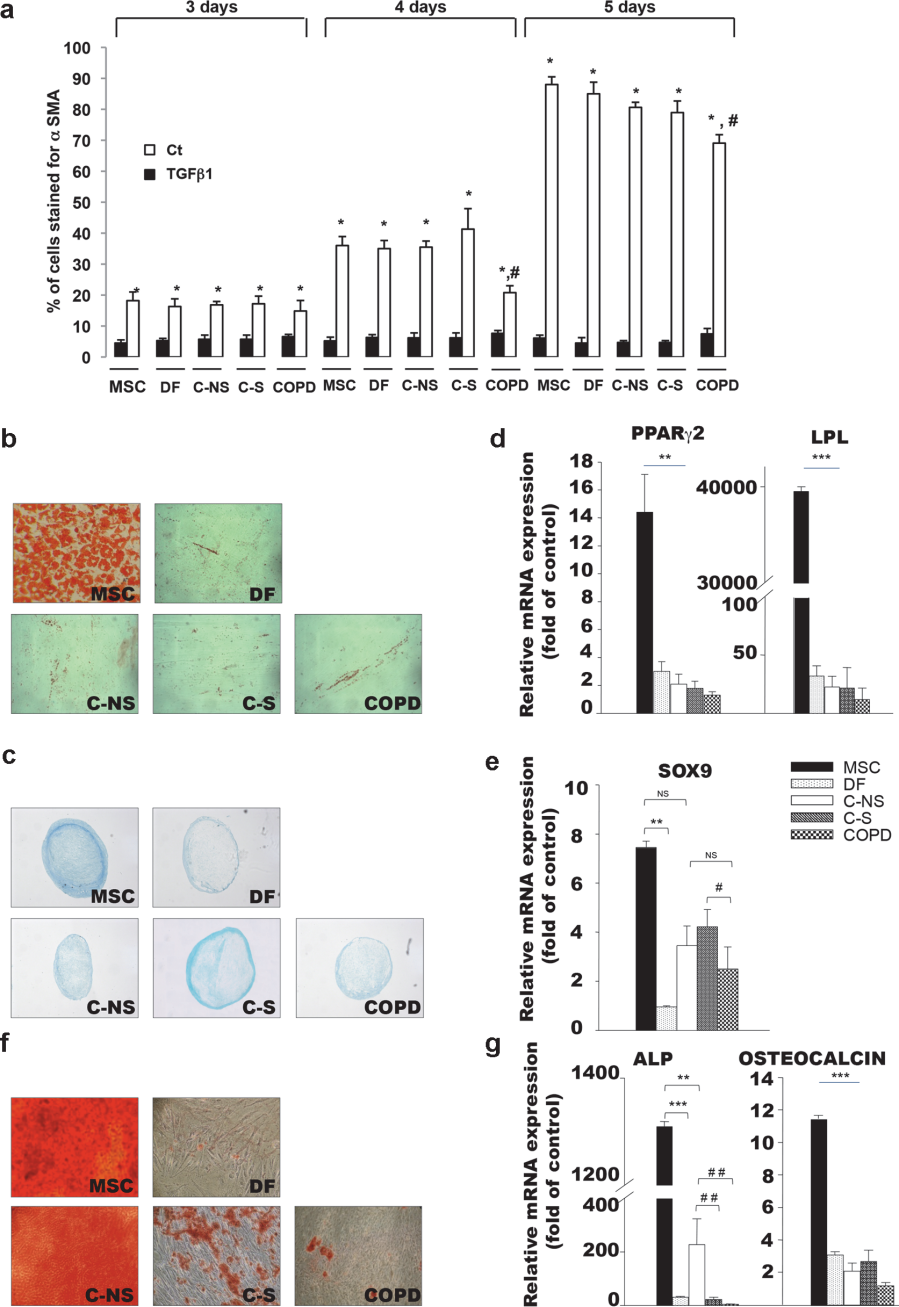
*In vitro* plasticity of lung fibroblasts. (a)Rate of lung fibroblast conversion into myofibroblasts after 3, 4 and 5 days exposure to TGF-β1. * p<0.05 compared to untreated cells. # p<0.05 compared to non- smoker (C-NS) and smoker controls (C-S) exposed to TGF-β1 for 4 or 5 days. (b) Oil-red O staining of, MSC, DF and lung fibroblasts from C-NS, C-S and COPD subjects12 days after exposure to adipogenic differentiation medium. (c)Alcian blue staining of MSC, DF and lung fibroblasts at day 24 following chondrogenesis initiation. (d,e)**(D, E)** Comparative real-time PCR analysis of mRNA expression of genes encoding for (d) adipogenic peroxysome proliferative activated receptor gamma 2 (PPAR-γ 2; early), lipoprotein lipase (LPL; late) and (e)chondrogenic Sox-9 marker. (f) Alizarin red staining of cells following 17 days of osteoblastic induction and (g)real-time PCR analysis of mRNA expression of osteoblastic alkaline phosphatase (ALP, early) and osteocalcin (OC, late) markers. (d,e,g) Results represent means ± SEM of at least 3 independent experiments performed with donor fibroblasts. NS: non significant; * p<0.05, ** p<0.01. MSC, mesenchymal stem cells; DF, dermal fibroblast; C-NS, non-smokers controls; C-S, smoker controls; COPD, smokers with COPD.

Having established that fibroblasts from the three groups of patients displayed similar basic characteristics, we first examined their ability to acquire an activated “myofibroblast” phenotype following treatment with transforming growth factor β1 (TGF-β1) for 3 to 5 days. Staining of α smooth muscle actin (α-SMA) was used to detect myofibroblasts and an increased number of positive cells was measured in all cell groups including in control dermal fibroblasts and MSC from day-3 to day-5 stimulation with TGF-β1 (Fig 2A). Nevertheless, after day-4 and day-5 TGF-β1 exposure, the rate of differentiation into myofibroblasts was significantly reduced in cells obtained from COPD patients (35.3 ± 1.8%, 40.4 ± 6.4% and 20.4 ± 2.4% for C-NS, C-S and COPD respectively at day 4 and 78.7 ± 0.5%, 80.9 ± 3.6% and 69 ± 2.4% at day 5 (Fig 2A), indicating a defective response towards TGF-β1 by COPD fibroblasts.

To determine whether lung fibroblasts shared the multilineage differentiation potential attributed to MSC, they were placed in adipogenic-, chondrogenic- and osteoblastic-conditioned media. Staining with Oil red O, alcian blue and alizarin red dyes combined to real time PCR assays for the transcriptional expression of lineage specific markers including peroxysome proliferative activated receptor gamma 2 (PPAR-γ2), lipoprotein lipase (LPL), Sox 9, alkaline phosphatase (ALP) and osteocalcin (OC) indicated that lung fibroblasts from C-NS patients were able to differentiate into adipocytes (or lipofibroblasts as some cells had spindle shape morphology), chondrocytes and osteoblasts more efficiently than dermal fibroblasts although their multilineage potential is significantly lower than that of control MSC (Fig 2B and 2G). In addition, fibroblasts from C-S or COPD subjects were found to differentiate with similar efficiency into adipocytes and/or lipofibroblasts and chondrocytes than C-NS ones (Fig 2D and 2E). Nevertheless, by comparison to C-NS fibroblasts, the ability to commit into osteoblasts was significantly diminished in C-S fibroblasts and was almost absent in COPD cells (Fig 2F and 2G). Overall, these results indicate that C-NS lung fibroblasts are multipotent and that this plasticity is gradually lost in cells derived from smokers and COPD subjects.

### Lung fibroblasts display MSC-like markers that are progressively altered in smoker non-COPD and COPD patients

**Fig 3:**
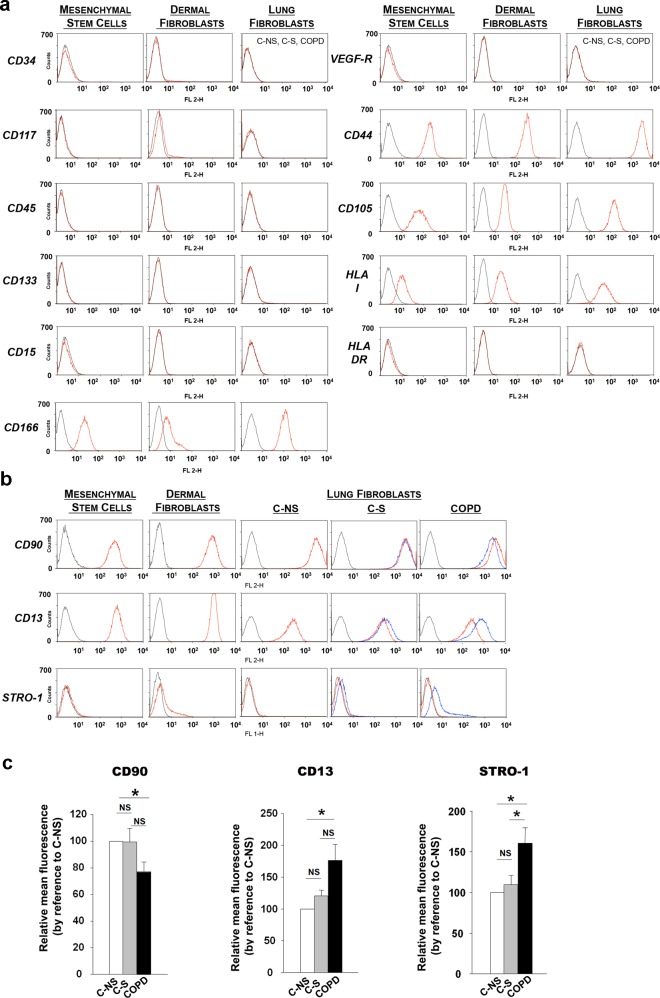
Cell-surface expression of MSC-associated markers. (a) Representative flow cytometry of CD34, CD117, CD45, CD133, CD15, CD166, VEGF-R, HLA-DR, CD44, CD105 and HLA-I cell-surface markers in mesenchymal stem cells, dermal fibroblasts and lung fibroblasts from non-smoker controls (C-NS), smoker controls (C-S) and COPD smokers (COPD). The dotted black line represents control immunoglobulin and the red refers to the specific marker. (b)Flow cytometry of CD90, CD13 and STRO-1. The dotted black line represents control immunoglobulins; the red line refers to the specific marker in non-smoker control fibroblasts (C-NS) and the blue represents smoker control (C-S) or COPD lung fibroblasts. (c) Histograms representing mean fluorescence variations for CD90, CD13 and STRO-1 between lung fibroblasts from the 3 groups (C-NS: n = 8, C-S: n = 8 and COPD: n = 9). NS, non significant, * p<0.05.

In line with observations already reported for MSC [26,37], flow cytometry analysis indicated that dermal and C-NS lung fibroblasts did not express CD34, CD117, CD45, CD133, CD15 (SSEA-1), VEGF-R2 (Flk-1) and HLA-class II molecules and were positive for HLA-class I antigens, CD44, CD105 and CD166 (Fig 3A). C-NS lung fibroblasts, like dermal fibroblasts, also expressed CD90 and CD13 but not STRO-1 (Fig 3B). Interestingly, these three latter markers were differentially expressed in C-S and COPD fibroblasts. Indeed, decreased level of CD90 and increased CD13 and STRO-1 markers were observed in fibroblasts from COPD patients (Fig 3B and 3C) and similar, but less pronounced phenotypic changes were found in C-S fibroblasts (Fig 3B and 3C). These results indicate that C-NS lung fibroblasts share with MSC the expression of a panel of cell surface markers and that this “immunophenotype” is progressively altered in fibroblasts from non-COPD and COPD smoker subjects.

### Immunosuppressive properties of lung fibroblasts are inhibited in smokers with or without COPD

**Fig 4:**
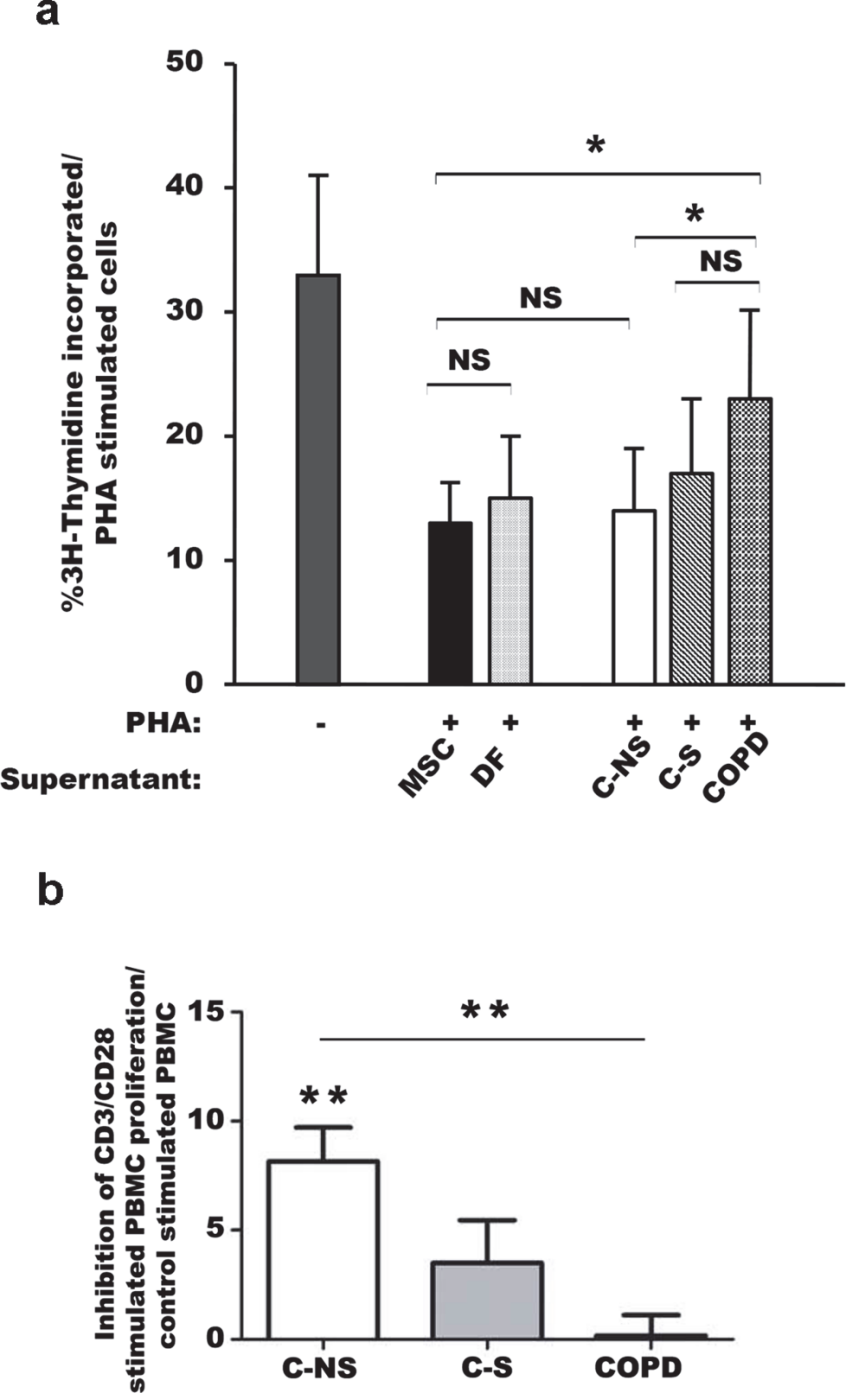
Immunosuppressive properties of lung fibroblasts. (a) Proliferation of phytohemagglutinin (PHA)-stimulated PBMC estimated ^3^H thymidine incorporation after 5-day exposure to supernatants collected from 24-h cultured MSC, DF and lung fibroblasts from C-NS, C-S and COPD subjects. (b) Proliferation of CD3/CD28 activated PBMC estimated by CFSE staining 4 days after exposure to supernatants collected from 24-h cultured lung fibroblasts. (a, b)NS: non significant, * p<0.05. MSC, mesenchymal stem cells, DF, dermal fibroblasts; C-NS: non-smokers controls; C-S: smoker controls; COPD: smokers with COPD. Results are expressed as means ± SEM of at least n = 3 independent experiments made with the 25 patient fibroblasts.

As MSC can suppress immune reactions through paracrine mechanisms [11], we also investigated this function in lung fibroblasts by measuring the proliferation either PHA- or CD3/CD28- activated PBMC exposed to fibroblast-derived supernatants. We found that like supernatants of MSC and dermal fibroblasts, those from C-NS fibroblasts inhibited PBMC proliferation while supernatants from C-S exhibited a mild but not statistically significant immunosuppressive effect and those from COPD patients were significantly ineffective (Fig 4A and 4B).

### Aberrant expression of Hh signaling components in non-COPD and COPD smoker patients

**Fig 5:**
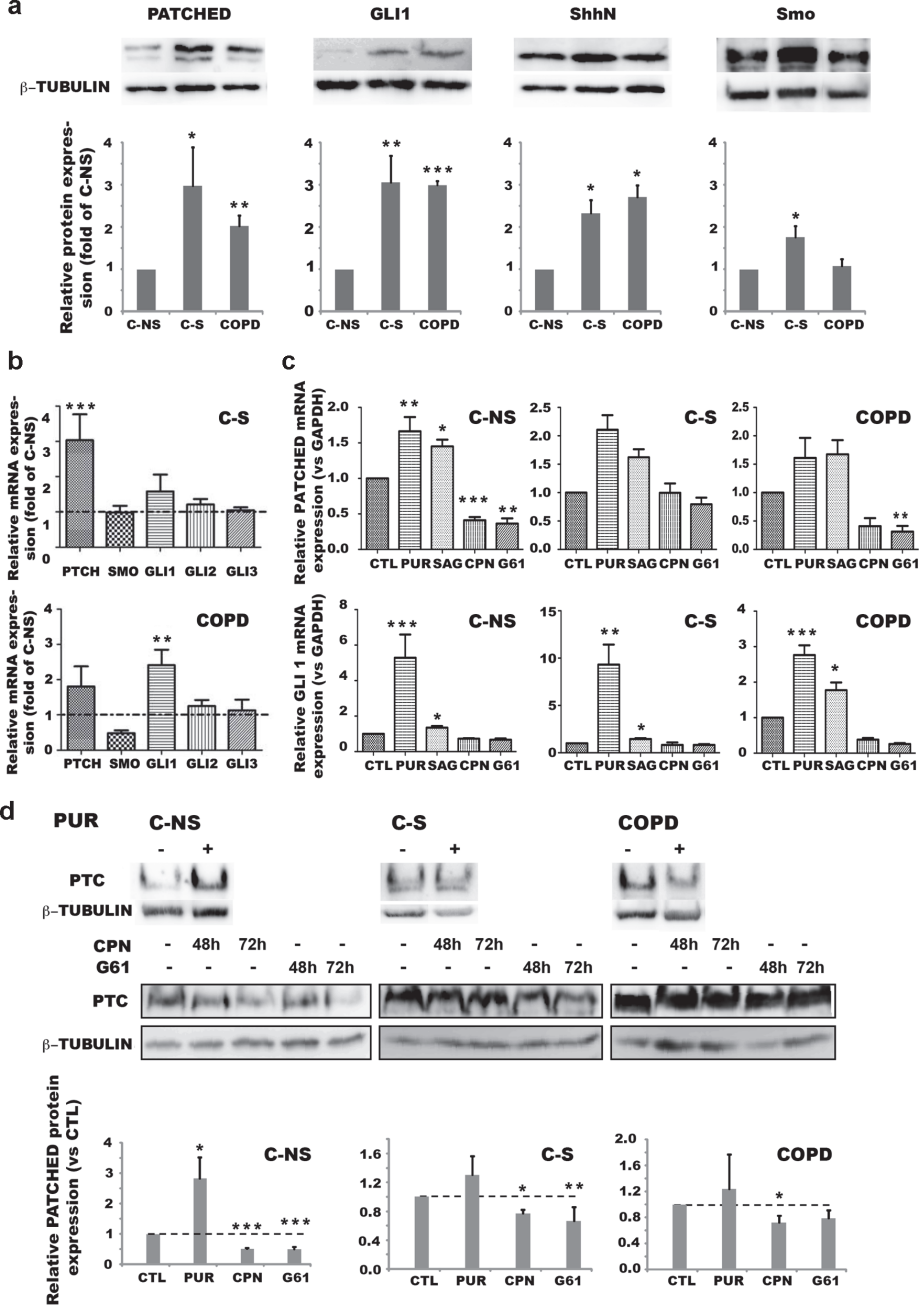
Over-activation of Hedgehog signaling pathway in C-S and COPD fibroblasts. (a)Upper panel: a representative western blot showing the protein expression of Patched, Gli1, ShhN and Smo in C-NS, C-S and COPD lung fibroblasts at molecular weights of approximately 140, 160, 20 and 100 kDa respectively which correspond to the expected MW of these proteins, β-tubulin was used as a loading control. Lower panel: histograms representing the mean of signal intensity of Patched, Gli1, ShhN or Smo for each patient group from at least 3 independent experiments. Protein expression was reported as fold of C-NS. (b) Transcriptional expression of Hh signalling pathway components in C-S and COPD lung fibroblasts compared to C-NS cells as assessed by quantitative real-time PCR analysis. (c)Quantitative real-time PCR analysis showing transcriptional expression of Patched (PTC) and Gli-1 in C-NS, C-S and COPD lung fibroblasts following exposure to DMSO (CTL), purmorphamine (PUR), SAG, cyclopamine (CPN) or GANT 61 (G61). (d)Upper panel: representative western blot showing the expression of Patched (Ptc) following 48h-treatment with pumorphamine (PUR), and 48h- or 72h- treatment with either cyclopamine (CPN) or GANT-61 (G61). β-tubulin was used as a loading control. Lower panel: relative western blot quantification of patched protein expression in C-NS, C-S and COPD lung fibroblasts following 48hour-exposure to DMSO (CTL), purmorphamine (PUR), cyclopamine (CPN) or GANT 61 (G61). (a, b, c, d)Results are expressed as means ± SEM of at least n = 3 independent experiments performed with at least n = 5 samples per fibroblast group. **(A, B)** * p< 0.05, ** p< 0.01, *** p< 0.001 compared to C-NS. **(D, E)** * p<0.05, ** p<0.01, *** p<0.001 compared to drug untreated CTL. C-NS: non-smokers controls; C-S: smoker controls; COPD: smokers with COPD.

In order to investigate potential mechanisms involved in the alterations of the stemness phenotype for fibroblasts derived from C-S and COPD patients, we focused on the Hh signaling pathway, which has been shown to control stem cell fate. We first measured the transcriptional and protein expression of components of the Hh pathway in C-NS, C-S and COPD lung fibroblasts and found that all three groups expressed Patched, Gli1, Sonic Hedgehog (ShhN) and the Hh transducing receptor Smoothened (Smo). However, higher levels of the Hh target genes Patched and Gli1 were detected in C-S and COPD fibroblasts compared to cells from C-NS subjects (Fig 5A and 5B), supporting the idea that Hh signaling is over-activated in C-S and COPD lung fibroblasts. Moreover, C-S and COPD fibroblasts produced higher amounts of activating peptide ShhN (Fig 5A), suggesting that increased ShhN release by C-S and COPD cells might be responsible for the up-regulation of the Hh pathway. Curiously, and in comparison with control C-NS fibroblast, the expression of the Hh transducing receptor Smo is enhanced in C-S fibroblasts but unaffected in COPD fibroblasts. In addition, we showed by immunocytochemistry that primary cilia were present in lung fibroblasts from the 3 groups suggesting that these cells can respond to SHH (S1 Fig). To investigate Hh signaling mechanisms in fibroblasts from C-S and COPD patients, we used modulators of the Hh pathway; the Smo agonists namely purmorphamine (PUR) and SAG, the Smo antagonist cyclopamine (CPN) and the Gli1 antagonist Gant61 (G61). While treatment with Smo agonists or antagonists enhanced or inhibited Gli1 mRNA expression respectively in the 3 groups of fibroblasts, it showed less effect on Patched mRNA and protein expression from C-S and COPD fibroblasts (Fig 5C and 5D). Indeed, while treatment with PUR or SAG induced up regulation of Patched in fibroblasts from 3 C-NS patients over 4, the up regulation of Patched is observed in the fibroblasts of only 1 C-S patient over 5 and 1 COPD patient over 4 (Fig 5D and data not shown). Moreover, CPN and G61 treatment induced lower down regulation of Patched in fibroblasts from C-S and COPD patients than in those of C-NS patients (Fig 5C and 5D). Our results show that fibroblasts from C-S and COPD patients present an aberrant activation of the Hh pathway. However, these fibroblasts seem to be less responsive to the Hh pathway modulators than fibroblasts from C-NS patients suggesting that an alternative non canonical Hh signaling independent of Smo and/or Gli1 occurs in C-S and COPD cells.

### Hh signaling is involved in the alteration of the stem cell phenotype of non-COPD and COPD smoker lung fibroblasts

**Fig 6:**
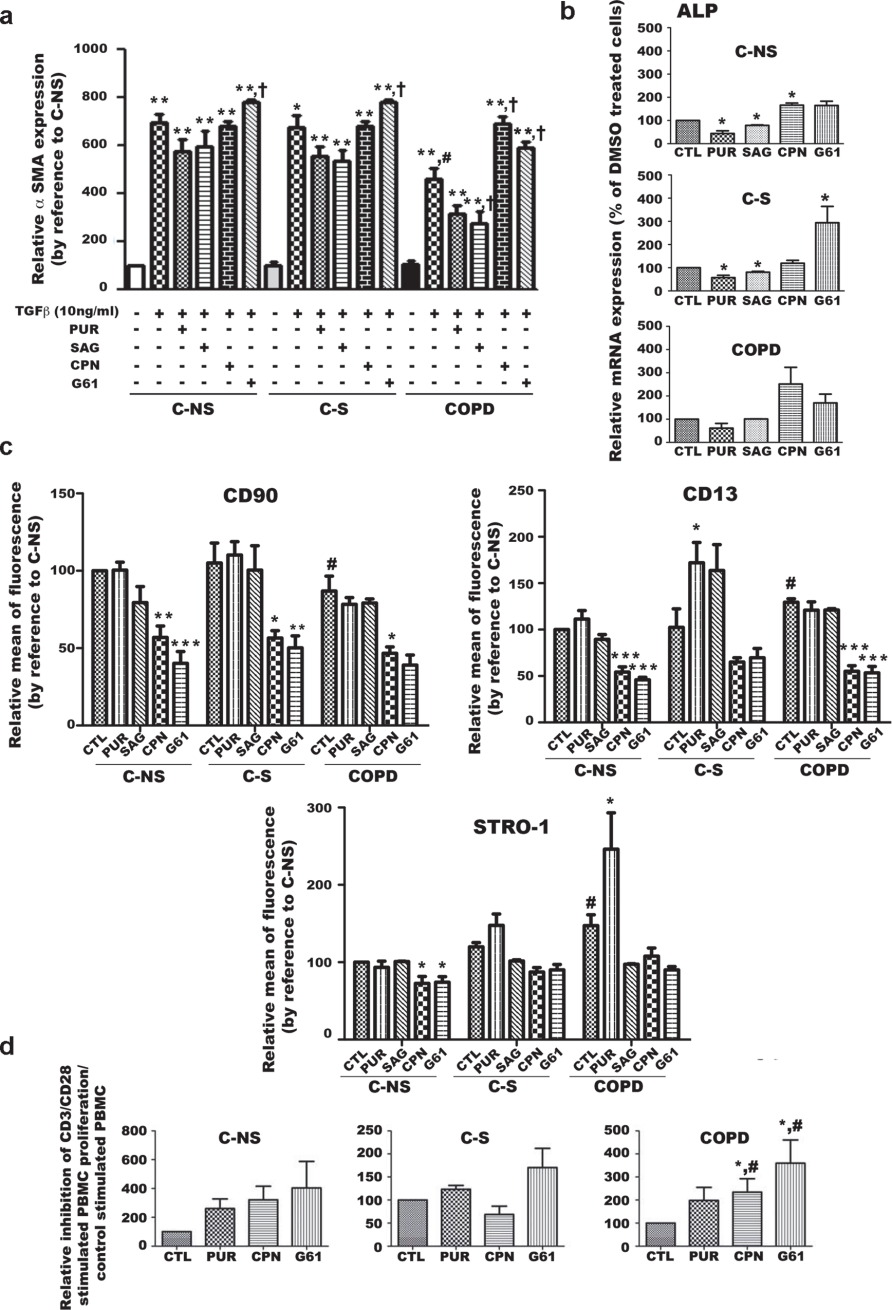
Impact of Hh pharmacological modulators on stemness properties of C-NS, C-S and COPD lung fibroblasts. (a) α-SMA immunostaining for myofibroblast differentiation of TGF-β-treated C-NS, C-S and COPD fibroblasts following incubation with purmorphamine (PUR), SAG, cyclopamine (CPN) or GANT 61 (G61). ** p<0.01 cells treated with TGFβ versus untreated cells; †p<0.05 cells treated with TGFβ and exposed to drugs versus cells treated with TGFβ alone; #p<0.05 fibroblasts treated with TGFβ from COPD patients versus from non smokers and smokers patients. (b) Quantitative real-time PCR analysis of transcriptional expression of alkaline phosphatase (ALP) to evaluate osteoblast differentiation of C-NS, C-S and COPD lung fibroblasts following exposure to DMSO (CTL), purmorphamine (PUR), SAG, cyclopamine (CPN) or GANT 61 (G61). * p<0.05 compared to CTL. (c)Flow cytometry analysis of cell surface expression of CD90, CD13 and STRO-1 in C-NS, C-S and COPD lung fibroblasts following exposure to DMSO (CTL), purmorphamine (PUR), SAG, cyclopamine (CPN) or GANT 61 (G61). * p<0.05 compared to C-NS, C-S and COPD treated with DMSO, respectively, # p<0.05compared to C-NS treated with DMSO. (d) Inhibitory effects of conditioned media from lung fibroblasts treated with DMSO (CTL), purmorphamine (PUR), cyclopamine (CPN) or GANT 61 (G61) on CD3/CD28 stimulated PBMC proliferation assessed by decrease of CFSE fluorescence. Results are expressed by reference to the respective control group. * p< 0.05 compared to CTL and # p<0.05 compared to PUR. **(A-D)** Results are expressed as means ± SEM of at least n = 3 independent experiments carried out using the 25 fibroblast cultures. C-NS: non-smokers controls; C-S: smoker controls; COPD: smokers with COPD.

The findings described above led us to postulate that a relationship might exist between the over-activation of the Hh signaling and the alteration in stemness of non-COPD and COPD smoker fibroblasts. Thus, we initially examined whether pharmacological inhibition or activation of Hh signaling affected the conversion of fibroblasts into myofibroblasts. Activation of the Hh pathway with PUR or SAG or its inhibition with CPN did not significantly TGF-β induced- α-SMA expression in C-NS and C-S fibroblasts while treatment with the Hh antagonist G61 increased the myofibroblastic conversion of these cells, probably through a non specific action (as this effect was not confirmed by CPN) (Fig 6A and S2 Fig). Conversely, activation or inhibition of the Hh pathway respectively decreased or increased the myofibroblastic conversion of COPD fibroblasts (Fig 6A and S2 Fig). In addition, we found that ALP transcriptional expression was inhibited by exposure to PUR or SAG and improved following treatment with CPN or G61, respectively in C-S and C-NS fibroblasts groups (Fig 6B), suggesting that conversion of fibroblasts into osteoblasts is sensible to Hh-signaling activation in these fibroblasts. However, these drugs did not statistically affect the osteoblastic differentiation potential of COPD cells suggesting that in COPD fibroblasts, non canonical Hh signaling predominates or that the canonical pathway is dysfunctional. On the other hand, we also observed that treatment with PUR improved STRO-1 expression in COPD fibroblasts but these results could not be confirmed with SAG suggesting that the effects of PUR on STRO-1 in COPD cells could be an artifact (Fig 6C). In addition, the cell surface expression of the CD90 and CD13 markers in these cells did not seem affected by these two agonists of the Hh signaling (Fig 6C).

Curiously, treatment with antagonists of the Hh pathway diminished cell surface expression of CD90, CD13 and STRO-1 in all lung fibroblasts, suggesting that down-regulation of Hh signaling could pertub lung fibroblasts immunophenotype (Fig 6C). Finally, exposure to purmorphamine, cyclopamine and Gant 61 did not significantly affect the paracrine immunosuppressive properties of C-S and C-NS fibroblasts while Hh antagonists significantly improved the ability of COPD cells to inhibit the proliferation of CD3/CD28 stimulated PBMC (Fig 6D). These results suggest that the Hh pathway controls the immunosuppressive properties of COPD but not C-NS and C-S fibroblasts, indirectly supporting the hypothesis that the Hh pathway is dysregulated in COPD cells.

## Discussion

The notion that lung fibroblasts play a key role in the development of COPD is now well accepted and supported by a series of publications describing the reduced capability of fibroblasts to sustain tissue repair in this disease [3,4]. However, because fibroblasts are generally considered as structural cells strictly devoted to maintenance of the extracellular matrix, most of the studies aiming at identifying dysfunction in COPD lung fibroblasts have only focused on these regenerative processes. Since fibroblasts from other organs have been shown to possess stem cells-like properties [8–11](we aimed in the present study to explore whether human lung fibroblasts may also display such features and whether COPD affects this phenotype. We further investigated the Hh pathway as a modulator of fibroblasts ‘stemness’ and as a signaling pathway that could be targeted by the disease. Thanks to a detailed characterization of parenchymal lung fibroblasts, our findings provide the first evidence that these cells indeed resemble MSC for several markers and that these properties are affected by chronic smoking exposure and COPD. Additionally, we identify Hh signaling as a pathway that is altered by tobacco smoking and is involved in the modulation of fibroblasts stem-like features. The hypothesis that lung fibroblasts could contribute to lung repair by adopting a MSC behavior [38,39] are supported by several lines of evidence describing that MSC confer protection against emphysema in mice chronically exposed to cigarette smoking [40] and promote lung repair in a murine model of bleomycin-induced lung injury [41]. Of interest, exposure to chronic cigarette smoke adversely affects MSC functions such as differentiation and migration potentials [40,42], indicating a susceptibility to smoking-induced damage shared by MSC and lung fibroblasts. Although replicative senescence was previously shown to promote loss of stem cell properties in MSC [35], this phenomenon cannot explain the phenotypic changes observed in our study, as C-S and COPD fibroblasts did not express senescence-associated markers. In addition, although we isolated fibroblasts from lung specimens of patients with cancer, it is unlikely that the phenotypic changes detected are associated with cancer since: *i)* controls and COPD patients suffered from similar cancer histological types; *ii)* biopsies were harvested at a distance from the tumor and were verified to be free of malignant cells and iii) primary cultures of lung fibroblasts did not exhibit CAF markers (data not shown and [7]). As the COPD fibroblasts included in our study were derived from patients with mild and moderate disease (according to the GOLD criteria), it would be interesting to determine whether the degree of alteration of stem cell like properties is more pronounced in fibroblasts from patients with severe COPD.

Our results also point to the idea that the loss in stem-like properties of fibroblasts is a gradual process induced by cigarette smoke that may begin in smokers and becomes more relevant with the development of COPD. This was particularly evident in the experiments assessing the differentiation of fibroblasts into osteoblasts, in the alteration of cell surface markers and in the inhibition of the fibroblast immunosuppressive properties towards PBMC, which were partially affected in non COPD smokers but considerably more in COPD smokers. The significance of these modifications in the pathophysiology of COPD is a crucial question that at present we can only attempt to address. For example, the changes in cell-surface expression we found in COPD fibroblasts have been previously documented in some MSC populations and were associated with decreased regenerative functions or increased tumorigenicity. In particular, the decreased positivity of CD90 was correlated with lung fibrogenesis [43] and impaired lung development [44], as well as loss of immunosuppressive activity [45] and cell-matrix adhesion and migration [46]. Furthermore, increased expression of CD13 and STRO-1 was associated with enhanced malignancy of MSC and emergence of cancer stem cells [47–49]. Furthermore, the failure in immunosuppressive activity of fibroblasts from smokers and COPD patients could be the critical factor in aggravating COPD because impaired fibroblast immunoregulatory properties may trigger the destruction of the pulmonary epithelium via chronic inflammation induced by tobacco smoking[1].

Another interesting observation reported by the present work is the aberrant activation of the Hh signaling in CS and COPD fibroblasts. Such an activation is probably a response of lung fibroblats to counteract the deleterious effects of tobacco smoking, as extensive activation of Hh occurs during repair of injured airway epithelium [13]. However, our observations showing that C-S and COPD lung fibroblasts poorly responded to Smo modulators suggest that canonical Hh signaling is defective or misregulated which could explain the failure in COPD to regenerate the lung or that canonical Hh pathway may not be the only Hh signaling mechanism in these cells. Indeed, Hh pathway activity resulting from mutational activation in the Hh signal transduction mechanism or ligand-independent aberrant signaling activation could be unresponsive to Smo or Gli1 modulators [50–53]. Despite the very low amount of patients included in the present study, our results are consistent with those reported by Domenech and coworkers (2012) on primary prostate fibroblasts as the authors showed that prostate fibroblasts which express Patched receptor did not significantly modified their expression of Gli and Patched following exposure to Shh ligands during 48 hours, suggesting that non canonical Hh pathway is activated in these cells.

Finally, our study provides strong evidence that the dysregulation of Hh signaling in C-S and COPD fibroblasts triggered some stemness alterations including the decreased myofibroblast differentiation, the reduced differentiation potential to osteoblastic lineage and the loss of immunosuppresive properties. Our data are consistent with earlier studies reporting that activation of Hh signaling inhibits the differentiation of MSC into osteoblast [54,55]. In addition, Shh has been reported to stimulate the proliferation of CD3/CD28 activated CD4+ T lymphocytes, emphasizing that increased release of Shh by COPD fibroblasts might potentiate Hh signaling to local T cells [56] and therefore contribute to the loss of their immune-suppressive properties. However and strinkingly, our data about myofibroblast differentiation and the impact of Hh signaling activation are in close discrepancy with the current litterature [57,58]. Indeed, we found that agonists or antagonists of Hh signalling did not affect the TGF-β-induced myofibroblastic conversion of C-NS and CS fibroblasts. This is probably due to the fact that TGF-β which was previously reported to activate the non canonical Hh signaling independently of Patched/Smo axis in lung fibroblasts [59], was used in high concentration in our experiments (10ng/ml instead 1ng/ml), thus bypassing the effects triggered by Hh signaling- agonists or antagonists. More difficult to explain are the effects observed following activation and inhibition of the Hh pathway in the TGF-β induced myofibroblastic differentiation of COPD fibroblasts. Nevertheless although non mechanistically elucidated, these effects reflect that Hh pathway is seemingly altered in COPD fibroblasts and that the non canonical pathway is likely predominant in these cells. Notably, our study is the first to report the status of the Hh pathway in lung fibroblasts as a function of smoking exposure and disease and to establish its relationship with the stem cell properties of fibroblasts. As aberrant activation of Hh signaling has been shown to be involved in the emergence and the maintenance of cancer stem cells [60], the immunophenotypic alterations together with the aberrant Hh signaling activation observed in lung fibroblasts from smokers, particularly those with COPD, may partially explain why chronic exposure to cigarette smoke affects lung regenerative properties and why smokers are more susceptible to lung cancer. However, future investigations will be needed to determine the mechanisms leading to Hh signaling dysfunction in non-COPD and COPD smoker lung fibroblasts and to fully understand the exact role of this pathway in tobacco-exposed lung and COPD disease.

In conclusion, our study reveals new intriguing characteristics of lung fibroblasts and a novel mechanism that might account for COPD initiation and progression. New insights in this field are expected to offer valuable information for the development of therapies in regenerative lung medicine.

## Supporting Information

S1 Fig Detection of primary cilia in C-NS, C-S and COPD lung fibroblasts. Staining with acetylated tubulin (red signal) showing the presence of primary cilia (arrowhead). Nuclei were counterstained with DAPI (blue signal). Scale bar 10μm.
(TIF)

S2 Fig Effects of TGFβ1 and SAG on the conversion of lung fibroblasts to myofibroblasts. Lung fibroblasts were treated with 10 ng/ml of TGFβ1 in presence of serum (a) or without serum (b). * p<0.05 compared to untreated cells. # p<0.05 compared to COPD fibroblasts and non- smoker and smoker controls exposed to TGFβ1 for 3 days.† compared to COPD fibroblasts treated with TGFβ1and SAG and COPD fibroblasts exposed to TGFβ1.
(TIF)
