# Supplementary figures and images for: Lung Fibroblasts Share Mesenchymal Stem Cell Features Which Are Altered in Chronic Obstructive Pulmonary Disease via the Overactivation of the Hedgehog Signaling Pathway
Source: PLoS One. 2015 Mar 27;10(3):e0121579. doi: 10.1371/journal.pone.0121579 (PMC4376723; doi:10.1371/journal.pone.0121579)

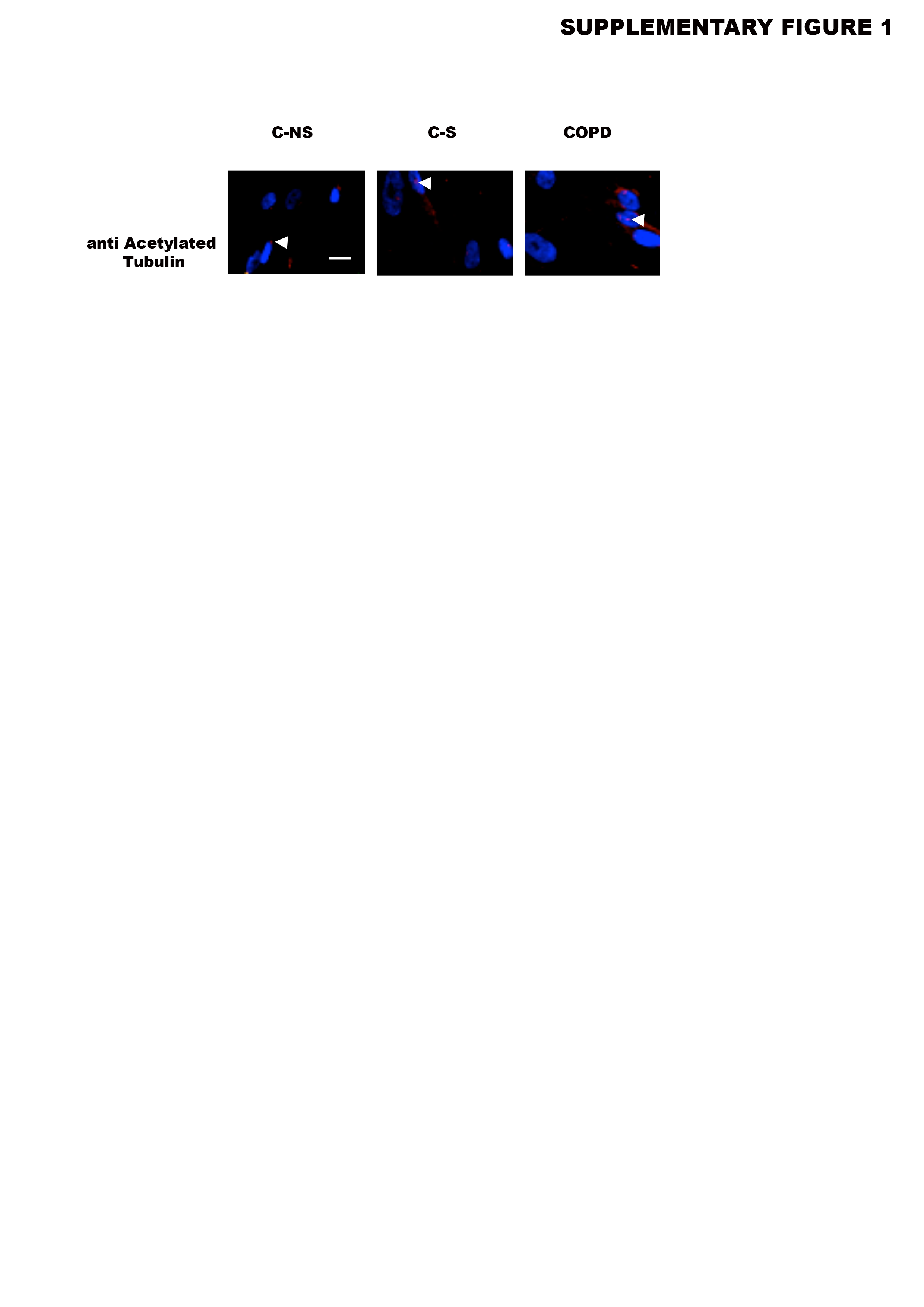

Supplement: S1 Fig — Staining with acetylated tubulin (red signal) showing the presence of primary cilia (arrowhead). Nuclei were counterstained with DAPI (blue signal). Scale bar 10μm. (TIF) [file pone.0121579.s001.tif]

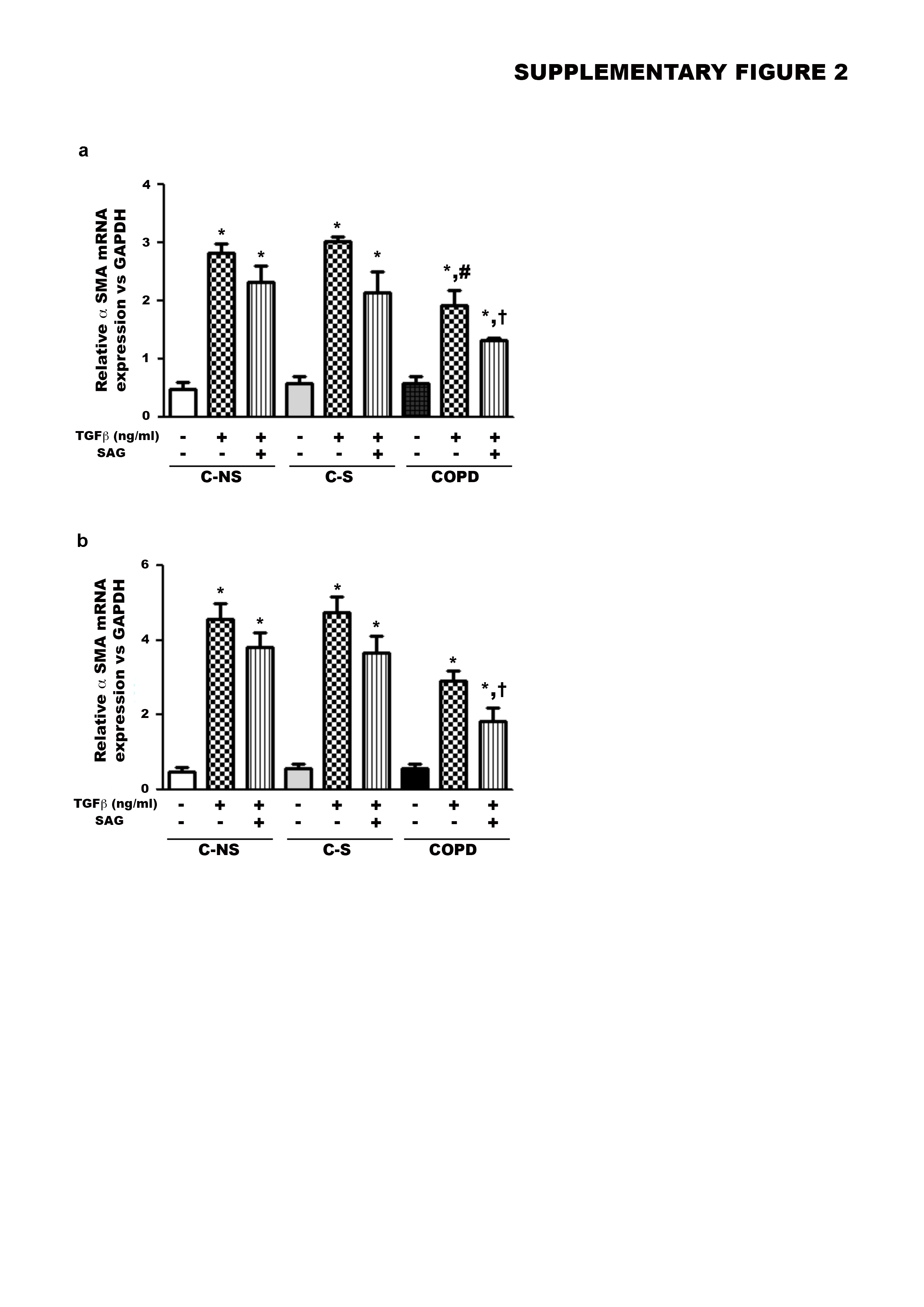

Supplement: S2 Fig — Lung fibroblasts were treated with 10 ng/ml of TGFβ1 in presence of serum (a) or without serum (b). * p<0.05 compared to untreated cells. # p<0.05 compared to COPD fibroblasts and non- smoker and smoker controls exposed to TGFβ1 for 3 days.† compared to COPD fibroblasts treated with TGFβ1and SAG and COPD fibroblasts exposed to TGFβ1. (TIF) [file pone.0121579.s002.tif]
